# Preparation for Endurance Competitions at Altitude: Physiological, Psychological, Dietary and Coaching Aspects. A Narrative Review

**DOI:** 10.3389/fphys.2018.01504

**Published:** 2018-10-29

**Authors:** Martin Burtscher, Martin Niedermeier, Johannes Burtscher, Dominik Pesta, Jiri Suchy, Barbara Strasser

**Affiliations:** ^1^Department of Sport Science, University of Innsbruck, Innsbruck, Austria; ^2^Austrian Society for Alpine and Mountain Medicine, Innsbruck, Austria; ^3^Laboratory of Molecular and Chemical Biology of Neurodegeneration, École Polytechnique Fédérale de Lausanne, Lausanne, Switzerland; ^4^Institute for Clinical Diabetology, German Diabetes Center, Leibniz Institute for Diabetes Research at Heinrich Heine University, Düsseldorf, Germany; ^5^German Center for Diabetes Research, München-Neuherberg, Germany; ^6^Faculty of Physical Education and Sport, Charles University, Prague, Czechia; ^7^Department of Epidemiology and Preventive Medicine, University of Regensburg, Regensburg, Germany; ^8^Medical School, Sigmund Freud University, Vienna, Austria

**Keywords:** acclimatization, time course, higher elevation, aerobic performance, athletes

## Abstract

It was the Summer Olympic Games 1968 held in Mexico City (2,300 m) that required scientists and coaches to cope with the expected decline of performance in endurance athletes and to establish optimal preparation programs for competing at altitude. From that period until now many different recommendations for altitude acclimatization in advance of an altitude competition were proposed, ranging from several hours to several weeks. Those recommendations are mostly based on the separate consideration of the physiology of acclimatization, psychological issues, performance changes, logistical or individual aspects, but there is no review considering all these aspects in their entirety. Therefore, the present work primarily focusses on the period of altitude sojourn prior to the competition at altitude based on physiological and psychological aspects complemented by nutritional and sports practical considerations.

## Introduction

Aerobic performance decline with increasing altitude is a long-known observation made by mountaineers and scientists as well. Horace Bénédict de Saussure (1740 – 1799) was probably one of the first who not only described reduced climbing performance and symptoms of mountain sickness at high altitude (Mont Blanc, 4,810 m) but also measured related changes in physiological variables like heart rate, respiration and body temperature. It was the Summer Olympic Games 1968 held in Mexico City (2,300 m) that forced scientists and coaches to cope with the expected decline of performance in endurance athletes and to establish optimal preparation programs (Pugh, 1967, 1969; Daniels and Oldridge, 1970; Mellerowicz et al., 1971). Shortly after the Olympics, a two to three day stay at altitude has been proposed as an appropriate acclimatization period for altitude competitions (Shephard, 1973). This period has been considered to be a compromise to allow sufficient adjustment of cerebrospinal fluid acid-base balance and to minimize plasma and stroke volume decrements but also to recover from the travel to the competition destination and potential symptoms of acute mountain sickness (Shephard, 1973). However, earlier and especially later studies came up with different recommendations for altitude acclimatization in advance of an altitude competition, ranging from several hours to several weeks depending on the altitude, the type of competition at altitude, the performance level of athletes, the training during preparation, etc. (Muza et al., 2010; Chapman et al., 2013; Foss et al., 2017). Although most endurance competitions are held at sea level or low altitude, similar events at moderate or high altitudes are becoming more and more popular, e.g., the road cycling championships in Bogotá in 1995 (2,640 m); the Olympic ski races of Torino in 2006 (1,509–2,800 m); the UCI Mountain Bike and Trials World Championships of Livigno in 2005 (1,800 – 2,000 m); and repeat events like soccer championships in La Paz (3,600 m); the Ladakh Marathon (3,500 m); the Khardung La Challenge - an ultramarathon (72 km) event, starting at near 4,000 m, reaching 5,370 m and ending at 3,504 m; the Pasco Marathon – officially the highest in the world – was accredited by the International Association of Athletics Federations (IAAF) and the Association of International Marathons and Distance Racing (AIMS) as the marathon (42 km) permanently held at high altitude (4,380 m); or ski mountaineering events like Patrouille Des Glaciers (up to 3,650 m), the Red Fox Elbrus Race (up to 5,600 m), and many others. In order to take account of this trend this review is aimed at serving as a reference for coaches and physicians faced with questions of optimal preparation for altitude endurance competitions. Focusses are primarily set on the period of altitude sojourn prior to the competition at altitude based on physiological and psychological aspects complemented by nutritional and sports practical considerations.

## Methodological Aspects

A literature search has been performed to identify original articles and reviews (1) dealing with the acclimatization process to moderate (1,500–3,000 m) and high altitudes (3,000–4,500 m) primarily considering the time course of changes in physiological and psychological responses to exercise and related changes in endurance performance of healthy individuals and/or athletes, (2) dealing with nutritional aspects when sojourning to and training at altitude for a sustained period (>1 week). The search for papers (English and German language) was performed within the following databases: Pubmed/Medline, Web of Science, Science direct, Scopus, and Sport Discus. The keywords altitude and/or hypoxia were used in various combinations with acclimatization, preparation, exercise, training, competition, performance, endurance, athletes, coaching, time course, diet, nutrition, physiology, psychology. Reference lists of articles were also reviewed to ensure that relevant studies were included. Studies demonstrating a plateau of physiological and performance changes during prolonged altitude sojourn and making reasonable efforts to avoid potential confounding with training effects have been preferentially selected for discussion. In addition, a viewpoint of an experienced coach adds to the evidence derived from scientific studies. Due to the complexity associated with large differences between type of sports and performance level of athletes we do neither extensively discuss training strategies at altitude nor the wide variety of potentially available hypoxic methods (Wilber, 2007; Millet et al., 2010). Note: Exposure to hypoxia may occur at real altitude (hypobaric hypoxia), in hypobaric hypoxia chambers, or in normobaric hypoxia rooms or tents or by breathing hypoxic air via face mask. Moreover, effect of erythropoietic responses was not a core focus of the present review due to the inconsistency of study findings and because it does not become relevant before 2–3 weeks at altitude and may therefore not play an important role for usual preparation for altitude competitions (Sawka et al., 2000; Millet et al., 2010; Płoszczyca et al., 2018). Novel and important features of the present review are the consideration of a large spectrum of factors influencing exercise performance, including physiological, psychological, nutritional, and practical aspects of optimal preparation for endurance competitions at altitude.

## Physiological Aspects of Acclimatization to Altitude and Related Changes in Performance

Many studies have evaluated effects of high-altitude exposure on exercise performance but only a few groups performed repeated testing in order to establish a time course of acclimatization and related changes in physiological parameters and performance. Studies differ particularly with regard to living altitude, duration of the altitude exposure, performance level of individuals, training characteristics and conditions. Therefore, characteristics of the acclimatization process are presented by selection of appropriate studies, which considered potential confounders like training effects and repeatedly determined a broad set of physiological variables and exercise performance at altitude. The validity of these findings is discussed based on the results of other altitude studies.

### Time Course of Acclimatization and Its Effects on Ventilatory and Cardiovascular Parameters and Exercise Performance at High Altitude (3,000–4,500 m)

**FIGURE 1:**
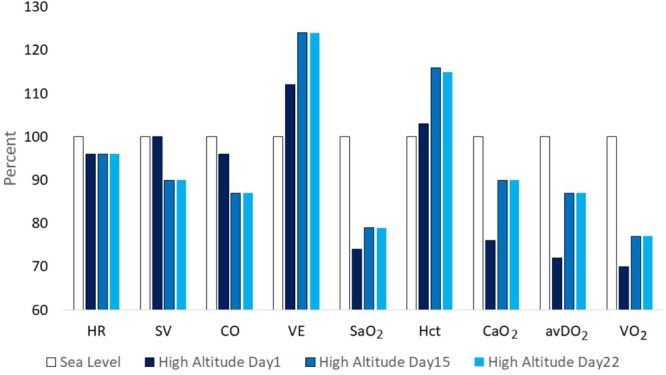
Changes of physiological parameters at maximal exercise from sea level (=100%) to day 1, 15, and 22 at altitude (4,300 m) (according to data from Horstman et al., 1980). HR, heart rate; SV, stroke volume; CO, cardiac output; VE, minute ventilation; SaO_2_, arterial oxygen saturation; Hct,: haematocrit; CaO_2_, arterial oxygen content; avDO_2_, arterio-venous oxygen difference; VO_2_, pulmonary oxygen uptake.

**FIGURE 2:**
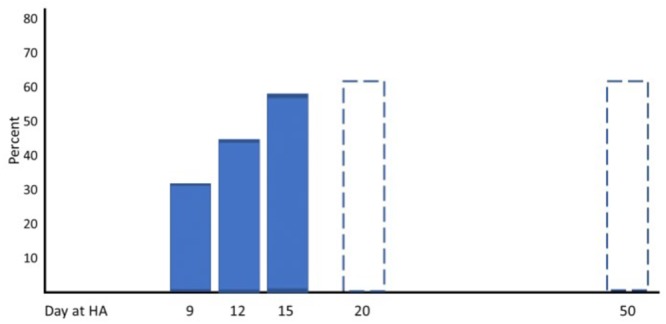
Percent improvement of submaximal performance from baseline at acute altitude with increasing duration of the high altitude (HA; 4,000 – 4,300 m) exposure (according to data from Buskirk et al., 1967 and Horstman et al., 1980).

Three well-designed studies from the 1960s and 1970s are chosen as examples to demonstrate typical changes of physiological responses to exercise accompanied by alterations in performance. Horstman and colleagues exposed 9 healthy men for 22 days to terrestrial altitude (4,300 m) (Horstman et al., 1980). Maximal and submaximal exercise tests were performed on a treadmill and a large set of physiological parameters was assessed at sea level (SL) on day 1 (HA1), day 15 (HA15), and day 22 (HA22) at high altitude. Maximal exercise ventilation increased steeply from SL to HA1 and further to HA15. A pronounced fall of arterial oxygen saturation (SaO_2_) occurred at HA1 which had partly recovered at HA15. These changes are the well-known consequence of ventilatory acclimatization to high altitude (hypoxia). Low levels of oxygen partial pressure (pO_2_) stimulate the hypoxic ventilatory response (HVR), increasing resting and exercise ventilation over time at high altitude, but also an elevated response to CO_2_ (hypercapnic ventilatory response, HCVR) may contribute to hyperventilation, counteracting the fall in SaO_2_ (Lenfant and Sullivan, 1971; Sato et al., 1992). There was a small decrease in maximal heart rate but a more pronounced reduction in stroke volume and maximal cardiac output from HA1 to HA15. These changes were accompanied by an increase of the haematocrit (Hct) due to haemoconcentration because of elevated diuresis at high altitude and the resulting reduction of plasma volume (Lenfant and Sullivan, 1971; Sawka et al., 2000). The increase of SaO_2_ and Hct improved the initial decrease of the arterial oxygen content (CaO_2_) contributing to improved oxygen delivery to working muscles, increased arterio-venous oxygen difference (avDO_2_) and maximal oxygen uptake (VO_2_max) at HA15 compared to HA1 (Figure 1). All physiological responses to maximal exercise remained nearly unchanged from HA15 to HA22, indicating that acclimatization was almost completed after 2 weeks at high altitude (Figure 1). Hyperventilation and haemoconcentration were most important responses during acclimatization but could only partly compensate for the arterial oxygen desaturation and the CaO_2_ reduction resulting in small VO_2_max improvements during prolonged high-altitude exposure (Horstman et al., 1980). However, submaximal exercise performance is more important for endurance competitions than maximal aerobic capacity. Thus, Maher and colleagues studied changes in submaximal endurance capacity (cycle ergometry) in 9 well-conditioned young men during a 12-day high-altitude (4,300 m) exposure (Maher et al., 1974). They did not see any change in VO_2_max from day 2 to day 12 but found a 45% increase in endurance performance (time to exhaustion). Time to exhaustion at the same workload was also determined thrice (day 9, 15, and 22) in the study by Horstman et al. (1980) demonstrating a 31 and 59% improvement on day 9 and day 15 without relevant change from day 15 to day 22 (Figure 2). This indicates a nearly linear increase of submaximal endurance performance during the first 2 weeks of altitude acclimatization. Buskirk et al. (1967) studied 6 members of a varsity track team during about 60 days of high-altitude (4,000 m) exposure. Cycling times during maximal exercise testing of the most well-trained participants indicate a pronounced recovery of endurance capacity during the first 2–3 weeks without essential changes over the remaining 30 – 40 days at altitude. Then cycle riding time and VO_2_max increased again (but not in the most well-trained athletes), in line with the observation from Klausen et al. (1966) who demonstrated such an increase after about 25 days at an altitude of 3,800 m (Klausen et al., 1966). These late improvements may be explained by individual training effects and/or erythropoiesis becoming effective after an about 3-week stay at altitude (Heinicke et al., 2005; Wehrlin et al., 2006). Such long exposures to altitude may be feasible for athletes to improve aerobic capacity for sea level performance or athletes only competing at altitude but not for those who have to compete repeatedly at altitude and sea level as well. Based on the presented data and more importantly on the time courses of physiological variables assessed during repeated maximal exercise testing at high altitude (Figure 1), an acclimatization period of at least 2 weeks seems to be necessary before being able to compete at the optimal level in a high-altitude endurance event. However, this time may not be sufficient for team sports regarding sprint ability at high altitude (3,600 m) (Buchheit et al., 2013; Girard et al., 2013). Finally, Fulco and colleagues evaluated six altitude and hypoxia pre-acclimatization strategies in order to minimize the development of acute mountain sickness and improve exercise during altitude exposure (Fulco et al., 2013). One of their key finding was that real altitude and hypobaric chambers were much more effective than the utilization of normobaric hypoxia.

### Time Course of Acclimatization and Its Effects on Ventilatory and Cardiovascular Parameters and Exercise Performance in Elite Athletes at Moderate Altitude (2,000–3,000 m)

Most endurance competitions are performed at rather moderate altitudes (2,000–3,000 m) than high altitudes (3,000–4,500 m). Whether the time course of acclimatization and performance recovery is different between moderate and higher altitudes cannot be assessed with certainty because there are only few comprehensive data sets available for moderate altitudes. Townsend and colleagues evaluated effects of a 2-week altitude training camp (2,700 m) in 7 elite cyclists (Townsend et al., 2016). After the 2-week period they ascertained elevated hypoxic chemosensitivity (increased HVR) associated with higher ventilation (VE), peripheral oxygen saturation (SpO_2_), and lower end-tidal carbon dioxide (pETCO_2_) values during exercise and improved cycling performance. These changes are well in agreement with those observed at higher altitudes (see above). Notably, the authors did not observe this correlation between VE, pETCO_2_ and SpO_2_ when exercising at sea level (Townsend et al., 2016). At sea level, endurance athletes typically show relatively low exercising VE because of a blunted respiratory controller gain, compared to non-athletes (Miyamoto et al., 2012). This adaptation is considered to be beneficial due to lower work of breathing and the not negatively affected blood flow to locomotor muscles by a respiratory metaboreflex (Scoggin et al., 1978; Harms et al., 1997; Dempsey and Wagner, 1999). When acutely exposed to moderate or in particular to high altitude, however, low exercise VE and reduced SpO_2_ may be detrimental to performance, which was demonstrated to be more pronounced in individuals with higher aerobic capacity (Ferretti et al., 1997; Burtscher et al., 2006). Hence, VE, SpO_2_ and performance may faster recover with acclimatization in highly trained individuals (Mellerowicz et al., 1971; Johnston and Turner, 1974; Burtscher et al., 2006). Not only the elevated HVR during a prolonged stay at high altitude but also increase of the HCVR (response to CO_2_) may contribute to exercise hyperventilation and prevention of pronounced SpO_2_ and performance decline (Sato et al., 1992).

Also the study by Schuler et al. (2007) demonstrated similar changes in physiological and performance parameters at 2,340 m as shown for higher altitudes. For instance, hematological parameters, VO_2_max values and times to exhaustion increased during 14 days at altitude, remaining essentially unchanged during the subsequent days. It has to be mentioned, that athletes in that study performed their training at low altitude but had about 19 h daily at 2,340 m. Interestingly, the authors reported a close relationship between changes in resting hemoglobin concentration (haemoconcentration) and time to exhaustion (Schuler et al., 2007). Since exercise values on ventilation and SpO_2_ are not shown, the impact of ventilatory acclimatization on performance changes cannot be evaluated. Nevertheless, an acclimatization period of at least 2 weeks seems also to be needed for competitions at moderate altitude as shown for higher altitudes. At higher altitude, performance improvements were associated with changes in exercise SpO_2_ levels but not with changes in haematocrit levels (Fulco et al., 2011), possibly indicating a more important impact of ventilatory acclimatization than haematocrit changes on exercise performance. These observations on performance recovery suggest that ventilatory acclimatization might be more important at higher altitudes and haemoconcentration (increase in haematocrit) rather at moderate altitudes.

The risk of high-altitude illness clearly increases with altitude and may be reduced by staging, i.e., reaching higher altitudes in 2 or more steps (Fulco et al., 2009). Fulco and colleagues evaluated the effect of staging (6 days) at 2,200 m on time trial (TT) performance (720 kJ cycle) at 4,300 m in healthy men (Fulco et al., 2009). They demonstrated a 44% improvement in the TT performance after 6 days at 2,200 m compared to acute exposure without staging. In contrast, living at lower altitudes, i.e., 1,520 m, did not improve VO_2_max or SaO_2_ in comparison to sea level residents when acutely exposed to high altitude (3,050 m) (Tucker et al., 1984) indicating that the altitude of residence, at least up to about 1,500 m, does not significantly influence the time course of acclimatization to high altitude. Chapman and colleagues showed that living at the altitude where the competition takes place is most favorable, whereas acclimatization to higher altitudes than the competition altitude will negatively impact on performance (Chapman et al., 2016).

### Time Course of Muscle Metabolic Adaptations and Related Exercise Performance at Altitude

The skeletal muscle phenotypic responses to a hypoxic stimulus is diverse and mainly depends on duration and degree of hypoxia. While chronic exposure of lowlanders to severe and chronic hypoxia is associated with a loss of muscle mass and mitochondrial function (Levett et al., 2012), skeletal muscle is able to adapt to subacute exposure to moderate altitude. Adaptations to moderate altitude are mostly characterized by different features of acclimatization rather than deterioration.

Green et al. observed a decrease of free adenosine diphosphate (ADP) in response to an exercise challenge at 4,300 m after 3 weeks of acclimation compared to the same challenge after acute ascent to this altitude, resulting in tighter metabolic control (Green et al., 1992). This apparently increased ADP sensitivity could be a compensatory mechanism to diminish excess production of reactive oxygen species (ROS), which occurs during hypoxic exposure (Moller et al., 2001). In line with this, previous observations revealed decreased ADP sensitivity in older individuals associated with increased mitochondrial ROS production (Holloway et al., 2018). In addition, 3 weeks of altitude exposure were also sufficient to induce a substantial reduction in glycolytic rate and lactate production during exercise, indicating that this time frame is sufficient to induce metabolic adjustments and allow acclimatization to moderate altitude (Green et al., 1992). Interestingly, this metabolic switch was not reflected at the level of skeletal muscle mitochondria as another study by Jacobs et al. (2013) revealed that the capacity for mitochondrial fat oxidation was unaffected upon exposure of lowland natives to 9–11 days of 4,559 m. When analyzing muscle mitochondrial function from biopsy samples of the vastus lateralis of healthy, young and physically active males, the authors reported no changes in coupling or respiratory control, indicating that qualitative and quantitative aspects of mitochondrial function were largely unaltered in response to subacute exposure to high altitude. However, the authors reported a tendency for a decrease of maximal oxidative phosphorylation capacity following acclimatization using the permeabilized muscle fiber approach. This slight decrement did not translate into diminished exercise performance as when oxygen supplementation was used during an incremental exercise test at high altitude to restore sea level conditions, maximal exercise capacity was not impaired. This is not surprising as it has been shown that mitochondrial respiratory capacity exceeds maximal oxygen delivery during maximal whole-body exercise (Boushel et al., 2011). A possible explanation for the slightly reduced oxidative phosphorylation capacity could be the impairment of tricarboxylic acid (TCA) – and oxidative phosphorylation-specific enzymes reported utilizing the same altitude of 4,559 m (Margherita Hut, Monte Rosa, Italy) in healthy male volunteers after a similar, slightly shorter exposure of 7–9 days (Vigano et al., 2008). Although isolated enzymatic modifications in muscle have been reported to occur in response to about 10 days of altitude exposure, the diminished expression of these enzymes did not seem to have a dramatic effect on skeletal muscle mitochondrial oxidative capacity. These findings are in line with observations made during an ascent to Everest base camp at 5,300 m. While short-term, 19 days exposure to this altitude did not result in mitochondrial loss, 66 days at altitude were associated with a decrease of mitochondrial density by 21% (Levett et al., 2012). Most likely, this process possibly involving the mammalian target of rapamycin- (mTOR) mediated down regulation of peroxisome proliferator-activated receptor gamma coactivator 1-alpha (PGC-1α) transcriptional activity and suppression of mitochondrial biogenesis serves the purpose to curtail production of damaging ROS when mitochondrial PO_2_ is low.

Of note, mitochondrial respiration of both complex I and II substrates was reduced following 28 days at 3,454 m utilizing the same approach of permeabilised muscle fibers while citrate synthase activity remained unchanged (Jacobs et al., 2012). In the same study, LEAK respiration as a proxy of mitochondrial uncoupling decreased in these participants after a month at moderate altitude, possibly indicating improved mitochondrial coupling ensuing better O_2_ efficiency. Even after 16 days of high-altitude hypoxia (5,260 m), an increased oxidative phosphorylation coupling control with a better utilization of mitochondrial respiratory capacity for ATP synthesis was observed (Chicco et al., 2018). In another study, well-trained cross-country skiers were subjected to 2 weeks of moderate altitude of 2,100 m while training at 2,700 m above sea level. Following altitude exposure, VO_2_max at sea level was unchanged while short-term performance was improved, most likely due to increased muscle buffer capacity (Mizuno et al., 1990). Competitive road cyclists where either training at a simulated altitude of 2,300 m or at sea level. When their work capacity was subsequently tested at sea level and altitude after 3–4 weeks of training, the increase was more pronounced in the altitude group. The between-group difference of work capacity was most prominent in the retest at altitude. The group that completed the training at altitude exhibited decreased exercise blood lactate concentration and glycolytic capacity (Terrados et al., 1988). If the training load is adapted during hypoxic training sessions in order to avoid the induction of a state of overreaching, the “live low–train high” concept or intermittent hypoxic training is possibly useful for preparing for competition at altitude. Intermittent hypoxic training can thereby act as an additional adaptive stimulus for the muscle tissue. Although some aspects of muscle mitochondrial capacity can be affected without changes in mitochondrial volume density, performance is not impaired at moderate altitude. With subacute altitude exposure, mitochondrial coupling efficiency can even be improved. Overall, the effects of hypoxic exposure on muscle tissue prior to competition at moderate altitude are expected to be small. There is, in fact, evidence that hypoxic training prior to competition is associated with benefits.

### The Influence of Mode of Exercise on Physiological Responses and Performance When Preparing for High-Altitude Competition

Acclimatization studies were primarily performed with runners (Buskirk et al., 1967; Pugh, 1967; Faulkner et al., 1968; Mellerowicz et al., 1971; Chapman et al., 2016; Diebel et al., 2017) and more recently also with cyclists (Schuler et al., 2007; Townsend et al., 2016) or team sport athletes (Gore et al., 2008; Garvican et al., 2014). In general, acclimatization processes are the same in athletes of various types of exercises and thus, time courses and effects of acclimatization may also be assumed to be comparable. However, as discussed by Townsend et al., differences between breathing patterns during intense running and cycling might differently impact on the effects of ventilatory acclimatization in runners and cyclists (Townsend et al., 2016). Larger tidal volumes and end inspiratory lung volumes were achieved during cycling than running (Tanner et al., 2014). This may more negatively affect VE and SpO_2_ during high-intensity running than cycling (Rice et al., 2000). Thus, more pronounced ventilatory limitation in runners may explain why some studies did not observe clear beneficial effects of altitude acclimatization on running performance (Faulkner et al., 1968). In addition, recovery of endurance and sprint performance may need different time periods at altitude (Buchheit et al., 2013; Girard et al., 2013).

### The Influence of Sex on Physiological Responses and Performance When Preparing for High-Altitude Competition

Performance changes during sustained exposure to moderate and high altitudes have mostly been studied in male athletes. Fulco and colleagues found that sex played hardly a role in explaining the large interindividual variation of maximal and submaximal exercise performance at various altitudes and for different time periods at altitude (Fulco et al., 1998). With regard to physiological responses, Bhaumik and colleagues demonstrated similar chemosensitivity in males and females exposed to altitudes between 2,100 and 4,350 m (Bhaumik et al., 2003). A similar time course of ventilatory acclimatization has been reported for women, not different between the luteal and the follicular menstrual cycle phase, compared to men (Muza et al., 2001). Moreover, no differences between sexes were found for cardiopulmonary responses to normobaric hypoxia (4,800 m) (Boos et al., 2016). With regard to haemoconcentration, haematocrit increased in both sexes by 1.5 points during 24 h of acute exposure to 2,500 m with an additional 0.2-point increase per 500 m gain in altitude (Beidleman et al., 2016). Whereas haematocrit continued to increase in men during the 7-day exposure to moderate and high altitude, it leveled-off after 5 days in women. Thus, if at all, only small differences exist between sexes regarding the extent and the time course of acclimatization.

### Alternative Models to Live High and Train High to Prepare for Altitude Competitions

To our knowledge, there is no well-controlled study comparing the effects between the classical live high and train high concept and other combinations, i.e., live high and train low or live low and train high, to prepare for altitude endurance competitions. However, based on the presented effects of acclimatization to altitude on performance and the fact that optimal performance is achieved after acclimatization to the altitude where the competition takes place, live high and train high is very likely the optimal concept (Chapman et al., 2016). Moreover, this assumption is supported by logistic aspects and reasons of familiarization with the conditions at the competition site. Hence, living at altitude with only short interruptions for some training sessions at lower altitudes may represent, when logistically possible, an effective alternative to the classical concept. For instance, Schuler et al. demonstrated similar benefits from such a program as known from the classical concept (Schuler et al., 2007). Whether athletes really benefit for competition at higher altitudes when intense training sessions are performed at lower altitudes has to be shown in controlled experiments. Moreover, intermittent exposures to hypobaric hypoxia may also represent an option to prepare for altitude training/competitions. Beidleman and colleagues demonstrated physiologic adaptations and improved time-trial exercise performance at altitude (4,300 m) after 7 days (4 h/day) of intermittent high-altitude (4,300 m) exposures, with or without exercise training during exposures (Beidleman et al., 2008). These researchers also showed improved muscular performance after 3 weeks of intermittent hypobaric hypoxia (4 h/day, 5 days/week) which was closely related to increased resting SaO_2_ post exposure (Beidleman et al., 2003). In contrast, no beneficial effects on endurance performance at altitude were found after 1 week of normobaric intermittent hypoxia exposures (2 h at rest plus two 25 min of exercise) (Beidleman et al., 2009).

When time for optimal altitude acclimatization is not available due to logistic reasons, short-term arrival strategies (2 – 14 h before the competition) might be a (sub-optimal) option (Chapman et al., 2013; Foss et al., 2017).

## Psychological Aspects of Acclimatization to Altitude and Related Changes in Performance

Psychological factors are believed to have a large impact on exercise performance. Confirmation for this notion can be found in a wide range of evidence levels ranging from anecdotes (e.g., Tuffey, 2000) to systematic reviews (McCormick et al., 2015). However, the body of research focusing on psychological factors remains scarce compared to physiological aspects or technical skills (Tuffey, 2000). The evidence regarding optimal psychological preparation for endurance competitions at altitude is even scarcer, where, to the best of our knowledge, no study has been conducted so far. However, some conclusions might be drawn from the impact of (moderate) altitude on psychological factors. In this section, we will focus on research addressing psychological aspects regarding endurance competitions *without* exposure to altitude as well as reported changes of psychological aspects due to (moderate) altitude and connect these findings to derive recommendations for an optimal preparation in terms of psychological factors. Research on psychological factors impacting exercise performance can be classified into two categories: the influence of situational factors on exercise performance (i.e., psychological factors that vary over time and depend on environmental influences/changes of the human organism) and the connection between exercise performance and personality traits (i.e., psychological factors, which are believed to be somewhat stable over time). Although there is some research on the connection between responses to altitude (acute mountain sickness) and personality traits (Missoum et al., 1992; Niedermeier et al., 2017a), altitude is considered an environmental factor which affects mainly situational psychological aspects. Therefore, the primary focus of this section is on situational factors.

### Situational Psychological Aspects

Without exposure to moderate altitude, the following situational psychological aspects have been studied with respect to endurance performance: cognitive functions, role of mood state in training and competition, overtraining syndrome/high levels of stress, use of psychological strategies.

Cognitive functions were shown to be associated with performance-related variables. Impaired cognitive functions were reported with difficulties to regulate exercise intensity and an appropriate pacing (Van Biesen et al., 2016). Several aspects of cognitive functions can be negatively influenced by altitude, e.g., detection of visual stimuli, short term memory, spatial memory, motor speed/precision, complex reaction time, decision making, cerebral function (Virues-Ortega et al., 2004; Ainslie et al., 2013). Although the effects become more relevant at higher altitude (i.e., 3,500 m and above), moderate altitude has more subtle effects, out of which some are detectable, e.g., increased complex reaction time at 1,500 m (Denison et al., 1966). Furthermore, the exercise to exhaustion during a competition might amplify the adverse effects on cognitive function (Moore et al., 2012). Similarly, adverse effects on performance due to acute mountain sickness as reported in Shukitt-Hale et al. (1991) might be increased, even though acute mountain sickness plays a minor role in moderate altitudes with a prevalence of 9% at 2,850 m (Maggiorini et al., 1990). Decision making was shown to be impaired with a riskier behavior in (simulated) altitude (Pighin et al., 2012; Davranche et al., 2016). Decision making is of special importance in the pacing process during an endurance competition (Hettinga et al., 2017). It should be considered that with riskier decision making at altitude, there is the danger of overpacing during the competition. However, the peak in risky decision making occurs during acute exposure (i.e., after 3 h) to hypoxic conditions and time in hypoxic conditions showed a risk-reducing effect on decision making (Niedermeier et al., 2017b).

Mood state (including positive/negative affect, arousal, state anxiety, and in a wider sense perceived effort) affects several domains of training and competition (Raglin, 2001): Firstly, exercise performance is associated to the level of arousal/state anxiety. In general, very high and very low levels of state anxiety seem to be unfavorable for good performance (Fazey and Hardy, 1988). However, according to the individual zones of optimal functioning model by Hanin, the optimal level of anxiety is independent of skill level and sport task, but individual for every athlete (Hanin, 1997). Higher state anxiety levels have been reported at higher altitude (Oliver et al., 2012b), which might have an impact also at moderate altitudes. As a consequence, the athlete might get out of the usual individual zone of optimal functioning at moderate altitude due to increased state anxiety. Secondly, negative affect is associated with lower endurance performance (Renfree et al., 2012). Altitude levels showed an adverse effect on mood states, i.e., persons reported to feel less vigorous and more fatigued at an altitude of approximately 3,000 m compared to 2,200 m (Shukitt-Hale et al., 1990). Research on the time course of these mood disturbances suggests a peak after 1 day of arrival at altitude and a return to baseline after 42–52 h (Shukitt and Banderet, 1988). In endurance competitions, where persevering pain discomfort is a central aspect, perceiving a higher level of fatigue during competition is a disadvantage for the athlete. There is evidence that carbohydrate (CHO) supplementation can lead to a decreased perceived effort of exercise performance at altitude (Oliver et al., 2012a). Therefore, CHO supplementation prior to the competition – beside the known physiological benefits (compare Section “Carbohydrate Requirements”) – can also result in positive psychological effects. Similar results have been found for the supplementation of caffeine at moderate hypoxia, i.e., approximately 2,500 m altitude (Smirmaul et al., 2017). Thirdly, mood state is used as an indicator for overtraining syndrome, where mood state was shown superior compared to physiological parameters (Raglin and Wilson, 2000). Prevalence rates of overtraining syndrome are reported to range around 10–15% in endurance athletes (Koutedakis and Sharp, 1998; Raglin and Wilson, 2000). Given the mood disturbance at altitude, this indicator might be biased due to altitude.

### Use of Psychological Strategies

Regarding the importance of psychological factors, it is not surprising that several psychological strategies exist to enhance endurance performance and for an optimal mental preparation of the athlete. The strategies include: association and dissociation, imagery training, self-talk, goal setting (Tuffey, 2000; Hatzigeorgiadis et al., 2011; McCormick et al., 2015), out of which association might be mostly influenced by altitude.

Association is a cognitive strategy, where athletes focus on the signals of the body including fatigue, pain, muscle soreness and use this information for pace regulation. In contrast, dissociation is a technique, where athletes use various means to distract themselves of the unpleasant sensations of the body. Means of distractions are ranging from listening to music, mentally constructing a house, to doing mathematical operations. Both approaches increased endurance performance with medium- to large-sized effect sizes (McCormick et al., 2015). Association is reported to be more effective in elite endurance athletes, whereas dissociation is effective in non-elite endurance athletes (Raglin and Wilson, 2000). While the technique of dissociation is not influenced by altitude, the aforementioned changes in mood states might be relevant in athletes using association. Sensations of higher fatigue in altitude (Shukitt-Hale et al., 1990) might lead to an incorrect pace regulation.

Imagery training (i.e., mentally simulating situations prior to the competition), self-talk interventions (i.e., “my legs are strong and powerful” prior to and during the competition), goal setting (prior to the competition) were also shown to be effective in enhancing endurance performance (McCormick et al., 2015). Imagery training might include the sensation of the environment at moderate altitude, e.g., mild hypoxia and/or lower temperature, to be as realistic as possible. Moreover, these techniques might be less influenced by altitude compared to association.

### Derived Recommendations

The following recommendations can be given from these considerations related to the preparation of an endurance competition at altitude in terms of psychological aspects: Firstly, an early arrival (at least 42–52 h prior to the competition) is preferable to avoid possible riskier decision making, mood disturbances and the related detrimental effects on endurance performance. An early arrival is also recommended on the basis of physiological aspects (Chapman et al., 2013). Secondly, a pre-exposure of the athlete to moderate altitude to study the athlete’s individual reaction in terms of cognitive functions and mood states is advised. Ideally, the pre-exposure takes place in the same or a similar environment and altitude as the competition. In cases, where this is not possible, a pre-exposure to normobaric hypoxia corresponding to the competition altitude might be used. Monitoring changes in mood state, e.g., using the Profile Of Mood States (McNair et al., 1971), during pre-exposure helps to get knowledge on the impact of altitude on mood. A pre-exposure might also be used for imagery training. Especially in competitions taking place in altitudes exceeding 3,000 m Richalet’s hypoxia sensitivity test (Richalet et al., 2006) can provide information on the athletes’ sensitivity of acute mountain sickness prior to the competition. Thirdly, CHO and/or caffeine supplementation might support to decrease the perceived effort during competition.

## Nutritional Aspects When Preparing for Endurance Competition at Altitude

Nutrition can have a major impact on the physiological adaptations associated with altitude training and competition. First, because satiety hormones are influenced by hypoxia, and in further consequence, appetite and energy intake are suppressed (Karl et al., 2018). The most important prealtitude consideration may be iron status (Constantini et al., 2017). For example, a low iron status prior to a high-altitude environment can quickly lead to iron depletion during altitude training so that hematological adaptations are compromised. However, it is not just the neuroendocrine system and the oxygen carrying capacity and thus performance that is affected, the immunity and host defense in athletes can also be affected. It has been shown that the risk of illness and respiratory infection is increased during altitude exposure (Walsh and Oliver, 2016). This may be due to direct effects of hypobaric hypoxic conditions accompanied by oxidative stress but may be also the result of inadequate nutrition and recovery (Ross and Martin, 2015). This chapter highlights nutritional aspects that may support preparation for endurance competitions at altitude. Key nutrition-related concerns include the need for iron, higher energy and fluid requirements, adequate protein intake for preventing body mass loss, and a higher demand for antioxidant-rich foods to maintain robust immunity. Based on studies at sea level, probiotic supplements may provide a practical means of enhancing systemic immune function in athletes during intense training.

### Adequate Iron Status

Iron is necessary for optimal erythropoietic adaptation to altitude exposure. Suboptimal iron status (i.e., ferritin < 30 ng/mL) may result from limited energy and iron intake, poor bioavailability, or increased iron demands due to high training loads, environmental factors (hypoxia-induced erythropoiesis, haemolysis, sweating), menstrual blood losses, and genetics (Pedlar et al., 2018). Therefore, iron status should be at a high level (ferritin > 50 ng/mL) before attempting altitude training (Clenin et al., 2015). For example, red cell volume did not improve following 4 weeks of moderate altitude exposure in iron deficient runners (mean ferritin: 15 ng/mL), suggesting that athletes require sufficient pre-altitude serum ferritin levels to support accelerated erythropoiesis at altitude (Stray-Gundersen et al., 1992). Based on a recent consensus statement of the International Olympic Committee, athletes should be checked for serum ferritin 8–10 weeks prior to altitude training or competition (Bergeron et al., 2012). However, it should be noted that ferritin as an acute phase protein is increased during inflammation and after intensive exercise. Thus, for a valid interpretation of iron status, several measures, including hemoglobin, hematocrit, the mean corpuscular volume and the additional measurement of soluble transferrin receptor and transferrin saturation, serum iron, and zinc-protoporphyrin should be performed (Larson-Meyer et al., 2018). Athletes with inadequate iron status may need supplemental iron as long as iron deficiency is present at doses between 40 to 60 mg per day in conjunction with 500 to 1000 mg of vitamin C (Clenin et al., 2015; Constantini et al., 2017). More aggressive dosages (up to 200 mg per day) appear to be necessary while at altitude, particularly in treatment of iron deficient athletes (Constantini et al., 2017). Several weeks before altitude training, athletes should be advised to increase dietary iron intake, particularly from haem iron sources like red meat and seafood with the addition of legumes and green vegetables. Long-term iron supplementation without a diagnosis of iron depletion may be harmful due to side effects, such as iron overload, and is not recommended (Zoller and Vogel, 2004). Moreover, excessive supplementation of iron is thought to increase oxidative stress and production of free radicals (Koskenkorva-Frank et al., 2013). This is of utmost importance in two ways: First, acute exercise leads to oxidative stress and secondly, at high-altitude an elevated level of oxidative stress is already present.

### Energy and Hydration Needs

Energy and fluid requirements are higher at altitude. Weight loss is a common phenomenon at altitude because of hypoxia-induced appetite suppression combined with an increase in basal metabolic rate (Butterfield et al., 1992). Although a daily energy deficit of about 10% may not reduce performance in the short-term as long as glycogen stores are maintained (Kechijan, 2011), severe and prolonged energy restriction at altitude negatively influences muscle mass regardless of protein intake (Berryman et al., 2018), which can compromise systemic immune function (Gleeson, 2013). Athletes should be informed that their appetite may not accurately reflect their true nutritional needs. High-energy-dense snacks between regular meals or training sessions can help to meet higher energy requirements associated with exercise at altitude (Ross and Martin, 2015).

Within the first days of acclimatization, athletes are at high risk of dehydration due to increased respiratory water loss by enhanced ventilation, increased urinary water loss, and an increase in basal metabolic rate. Thus, athletes should be encouraged to drink sufficient fluids while at altitude. Experts recommend a daily fluid intake of 3–5 L to maintain fluid balance (Butterfield, 1999; Friedlander et al., 2008), best in the form of isotonic carbo-electrolyte drinks and juices (Yanagisawa et al., 2012), others suggest fluid needs as high as 7 L per day in cyclists during altitude training (Michalczyk et al., 2016). Importantly, drinking tea at altitude does not compromise hydration when consumed by regular tea drinkers, but it does have a positive effect on mood (Scott et al., 2004). Overall, authors recommend regular monitoring of body mass and urine osmolality during altitude training to ensure proper hydration and to prevent overdrinking since both hypohydration and hyperhydration impair performance and present a risk to health (Maughan and Meyer, 2013).

### Carbohydrate Requirements

At altitude, the stress response to exercise is enhanced, and thus CHO requirements are higher than at sea level (Katayama et al., 2010). CHO consumption before exercise in hypoxia can mitigate some of the negative symptoms of high altitude, like less oxygen saturation and ventilation (Golja et al., 2008). Barnholt and colleagues demonstrated suppressed endocrine responses, e.g., fasting glucose, insulin, epinephrine, and thyroxine, in caloric restricted subjects exposed to hypoxia (Barnholt et al., 2006). These authors suggested that such suppression may help to preserve energy stores to the detriment of oxygen delivery and the use of oxygen-efficient fuels. Another study found no differences in the endocrine response to hypoxia between sexes, but metabolic substrates (higher glucose and lower lactate levels in females) were still affected (Sandoval and Matt, 2002). These authors proposed that the lower absolute workload and/or the presence of estrogen in women might explain the observed metabolic differences. Moreover, carbohydrate utilization was shown to be reduced in women compared to men exposed to high altitude, probably explained by estrogen and progesterone concentrations in women (Braun et al., 2000). In addition, during deep sleep, hormone release of the hypothalamamic–pituitary–adrenocortical system is inhibited, while the release of growth hormone and prolactin is increased (Van Cauter et al., 2008). Thus, sleep disturbances at altitude might contribute to the catabolic effect of hypoxia.

During intense training sessions at altitude, CHO intake can increase up to 80% of total calories per day (Brouns et al., 1989). Since a reduction in blood glucose levels is linked to an increased immune activation, the ingestion of CHO during and immediately after exercise is of utmost importance, especially for athletes training at altitude (Nieman and Mitmesser, 2017). Based on current evidence, experts recommend 8–12 g of CHO per kilogram of body mass per day for daily fuel needs and recovery (Burke et al., 2011), additionally 30–70 g CHO per hour of exercise depending on exercise intensity and duration (Nieman and Mitmesser, 2017). For ultra-endurance events, the recommendation is higher at about 90 g per hour of multiple transportable CHO, ingested as a glucose:fructose drink in 2:1 ratios (Jeukendrup, 2014). However, recent evidence suggests that training sessions with reduced CHO availability (‘train-low’) may promote molecular adaptations and endurance performance but should be undertaken in conjunction with sessions with normal or high CHO availability during preparation for endurance competition (Bartlett et al., 2015; Marquet et al., 2016). In normobaric conditions, the results from Hansen et al. (2005) and Yeo et al. (2008) have shown that short-term training (3–10 weeks) in which about 50 percent of sessions are commenced with low muscle glycogen levels promotes training adaptations (i.e., increases the activities of enzymes involved in energy metabolism and mitochondrial biogenesis) to a greater extent than when all sessions are undertaken with normal or elevated glycogen stores. However, the train-low strategy should be considered only before traveling to altitude and should be avoided in the days leading up to altitude. Although reduced oxygen can inhibit mTOR (Liu et al., 2006), possibly contributing to muscle deterioration during hypoxia (Debevec et al., 2018), leucine supplementation did not prevent loss of fat-free mass during a 13-day trek to Everest Base Camp in a double-blind randomized study (Wing-Gaia et al., 2014). It needs to be established whether leucine directly interacts with mTOR during hypoxic conditions, which could attenuate loss of fat-free mass during longer duration high altitude exposure.

### Protein for Lean Body-Mass Retention

Weight loss and body composition changes are an unfortunate consequence of sustained hypobaric hypoxia, with lean body mass (LBM) comprises approximately 60–70% of body weight loss during high-altitude (>5,000 m) exposure (Wing-Gaia, 2014). Possible mechanisms of altitude-induced muscle wasting include downregulation of muscle protein synthesis and suboptimal energy and protein intake. Thus, it appears reasonable that a higher protein diet at altitude may be useful to improve retention of LBM. However, increasing protein intake may be problematic in two ways: on the one hand because of its high thermic effect (Westerterp and Kayser, 2006), on the other hand because of its satiating effect, although a higher protein diet appears to have no impact on appetite suppression at high altitude (Karl et al., 2018). As a consequence of low energy intake at altitude, athletes can benefit from consuming a low volume protein supplement high in branched-chain amino acids, especially leucine, to improve muscle protein synthesis and retention of LBM (Phillips and Van Loon, 2011; Wing-Gaia et al., 2014). The recommended protein intake for athletes engaged in heavy training at sea level are between 20 and 25 g of high-quality protein consumed after exercise (Witard et al., 2014), although athletes at altitude will require somewhat more protein. Based on a recent study, consuming a higher-protein diet (2.0 g protein/kg body weight per day) did not protect LBM during severe energy deficit at high altitude (Berryman et al., 2018). However, caution is warranted to extrapolate results from high to moderate altitude environments.

### Antioxidant Supplements

It has been shown that antioxidant status is impaired under hypoxic conditions and even remained impaired for 2 weeks following an altitude training camp (Pialoux et al., 2010). Although low oxygen pressure seems to be favorable to low ROS production, high altitude exposure can lead to enhanced ROS generation due to up-regulation of the mitochondrial electron transport chain, xanthine oxidase, and nitric oxide synthase (Dosek et al., 2007). High-altitude training appears to weaken both the enzymatic and non-enzymatic antioxidant systems (Quindry et al., 2016). During altitude training, cells release large amounts of ROS, which may lead to an imbalance between antioxidants and prooxidants (= oxidative stress), which may be prevented by additional intakes of vitamins and minerals. While antioxidant supplementation did not affect markers of oxidative stress associated with increased energy expenditure at high altitude (Subudhi et al., 2004), and may even interfere with adaptations to exercise at sea level (Paulsen et al., 2014), it is still unclear whether antioxidant administration inhibits adaptation to altitude training. Recent data within the UBC-Nepal expedition show that an oral antioxidant cocktail (vitamins C and E and α-lipoic acid) at clinically relevant doses did not alter cerebrovascular function and blood flow at sea level or after 12 days at high altitude (Hansen et al., 2018). However, it was proven that doubling the intake of antioxidant-rich foods was well tolerated and did not negatively influence the adaptive response to altitude training in elite endurance athletes, but increased hemoglobin levels (Koivisto et al., 2018). Moreover, dietary antioxidants can slow down the production of Th1-type cytokine interferon-gamma and in further consequence tryptophan breakdown by the enzyme indoleamine 2,3-dioxygenase (Strasser et al., 2016c). Tryptophan (TRP) represents as a precursor for serotonin production a key element for brain functioning. Hence, nutrients rich in TRP and antioxidants can have a positive impact on mood and sleep quality (Halson, 2014). In addition, diets high in CHO may increase brain TRP by insulin stimulation of large neutral amino acids into skeletal muscle, which results in an increase in free TRP (Fernstrom and Wurtman, 1971). Recently, we demonstrated that intense aerobic exercise is associated with TRP catabolism in trained athletes, which may play a role in the onset of central fatigue (Strasser et al., 2016a). Furthermore, weight loss is accompanied by a decline in TRP, and lowest TRP levels were observed in individuals with the lowest energy intake (Strasser et al., 2015). In the situation of heavy exercise at altitude in addition to reduced food intake, athletes may be faced with low levels of serotonin and this can influence an individual’s sensation of fatigue and may affect performance (Meeusen, 2014).

### Probiotics and Vitamin D to Prevent Infections

Prolonged intense exercise is associated with a transient depression of immune function and a heavy schedule of altitude training and competition can lead to immune impairment in athletes. This is associated with an increased susceptibility to upper respiratory tract infection (URTI) (Walsh and Oliver, 2016). Some studies have established that probiotic intake can enhance resistance to URTI in athletes as well as reducing gastrointestinal discomfort problems, a common problem in endurance athletes (Gleeson, 2016). Probiotics could possibly have beneficial effect in preventing such symptoms in these individuals by improving gut barrier function (Colbey et al., 2018). These positive clinical consequences provide evidence for the beneficial effects of daily probiotic ingestion in highly physically active people and may be attributable to modulation of the gut microbiota, the mucosal immune system and lung macrophage and T lymphocyte functions (Pyne et al., 2015). Some of these effects appeared to be connected with alterations in TRP metabolism (Strasser et al., 2016b).

Despite the increased UVB radiation from sunlight, vitamin D supplementation should be considered in athletes who stay at high altitude as vitamin D may influence iron metabolism and thus erythropoiesis (Smith and Tangpricha, 2015). In a study on vitamin D in mountaineers, the authors observed a significant decrease of serum vitamin D level after a 2-week high-altitude climb which was associated with modulation of immune processes (Kasprzak et al., 2015). Reduced dietary uptake of vitamin D or clothing could have contributed. Recent evidence supports an optimal circulating 25(OH)D of 75 nmol/L to prevent URTI and enhance innate immunity and mucosal immunity and bring about anti-inflammatory actions through the induction of regulatory T cells and the inhibition of pro-inflammatory cytokine production (He et al., 2016).

### Nutritional Recommendations

In summary, physiological adaptations that occur with training at altitude can be positively influenced by appropriate nutrition. In the case of an endurance competition at moderate or high altitude, the diet in the weeks prior to altitude exposure is of utmost importance, especially from the perspective of improving iron status and overall health. Table 1 provides dietary strategies to help optimize the preparation for endurance competitions at altitude.

**Table 1:** Dietary strategies to help optimize the preparation for endurance competition at altitude.

Increase iron intake (meat, fish) with the addition of legumes and green vegetables. Consider enhancers (vitamin C) and prevent inhibitors (coffee, black tea, calcium) of iron uptake.
Ensure a balanced energy balance. High-carbohydrate, nutrient-rich snacks are a good additional energy supply between regular meals, especially for those athletes who have a suppressed appetite.
Increase daily CHO intake to 10–12 g of CHO per kg of body mass. Additionally, 60 g up to 90 g CHO per hour of intense exercise is recommended as a glucose:fructose drink in 2:1 ratios. Commence some of the training sessions with sub-optimal CHO reserves (‘train-low’).
During the early phase of acclimatization, increase fluid intake to 3–5 l to ensure proper hydration. Use sport drinks instead of water. Drinking tea may improve mood in tea drinkers.
After exercise consume 20–25 g of high-quality protein to maximally stimulate muscle protein synthesis. Protein supplements containing leucine may reduce muscle wasting at altitude.
Small doses of tryptophan (1 g) may improve both mood and sleep quality. Foods high in tryptophan are nuts (cashews, walnuts, peanuts, almonds), sesame and pumpkin seeds, soybeans and grains.
Sufficient intake of antioxidant-rich foods should be preferred over supplements. However, evidence suggests that daily doses of >200 mg vitamin C may prevent athletes for common cold.
Probiotic supplementation at least 14 days before altitude training or competition with a daily dose of ∼10^10^ live bacteria may reduce the risk of respiratory and gastrointestinal illness.
Consume a 1000 IU/day vitamin D3 supplement in the autumn-winter months. At altitude, increase supplementation up to 4000 IU/day. Some foods such as oily fish (e.g., tuna, mackerel, salmon) and shiitake mushrooms are a good source of vitamin D3.

## Preparation for Endurance Competitions at Altitude From a Coaches’ Point of View

The optimum for achieving better results in endurance competitions at altitudes is to be born or at least live permanently and train at such altitudes (Fulco et al., 2007), or to move to altitude at any time over the course of the sporting carrier. With regard to sea-level performance, live high (at natural altitude) and train low seem to be the most promising protocol in athletes (Bonetti and Hopkins, 2009). In this context, the trend of endurance athletes moving to altitude has been confirmed by a number of case reports published for example at the United States Olympic Committee conference (Wilber, 2001; Suchý et al., 2009).

If for logistical and/or socioeconomic reasons athletes cannot move permanently to a higher altitude, it seems optimal to undergo an at least 2- to 4-week training camp before an important competition. The specific preparation cannot be done successfully without prior repeated testing in training camps at altitude to learn the course of acclimatization of the individual athlete. Moreover, several recent studies also report VO_2_max and TT improvements after using intermittent normobaric hypoxia protocols (Dufour et al., 2006; Czuba et al., 2011, 2018).

However, athletes mostly compete at low altitudes with only rare competition events at higher altitudes. In such matters, the preparation period for the altitude competition depends on the time interval between competitions, the importance of the altitude competition, and prior individual experiences and may vary between some hours and about 2 weeks. In more recent time, coaches and athletes are also faced with the opportunity to use artificial altitude (normobaric hypoxia chambers) for preparation purposes offering multiple combinations of artificial and real altitude exposures with and without training.

Coaches learned that the training camp should take place at the same altitude but in a different place than the planned competition. The reasons for training at a different place than the competition site are the same as at low altitudes: limiting the stay at the site of the competition. Even just the nomination for elite competitions, and especially the stay at its site, generally have considerably stressful effects on athletes. Moreover, the training options there are also often limited (Suchý et al., 2009).

For the first to sixth day of a 3-week preparatory training camp for example, the training must take into account the problems of one’s first days at altitude. Training should take place at a reduced load intensity (up to 75% of the maximum) and 60% of volume compared to similar training at normoxia. On approximately the fifth day it is necessary to redefine the current individual values of load intensity, which differ markedly from those used at normoxia. Verifying these intensities is of considerably greater relevance than at lowlands (Wilber, 2001; Suchý et al., 2009).

In order to accelerate the regeneration processes at altitude, inhalation of oxygen of lowland concentration can be used (via a mask or tent) (Wilber, 2001; Chapman et al., 2010). It is also important for successful handling of altitude for athletes to be mentally prepared for the fact that training at higher altitude will be much more demanding compared to low altitudes. Hypoxic training before going to altitude was suggested as a method potentially improving the ability to tolerate discomfort at altitude and thus improve exercise performance (Álvarez-Herms et al., 2016). Importantly, training intensity must be modified with regard for the given environment. The athlete should understand the essence of adaptation to an environment of hypoxia and the logic of modifying the training program.

From the 7th to 12th day the load can gradually be increased, but the rising intensity must be carefully monitored. If the athlete manages the first 14 days of the sojourn according to the plan, it is possible to gradually start shifting to the training customary at low altitudes starting with the third weekly microcycle, including segments at racing pace. Over the course of sojourns at higher altitudes there are generally three basic critical periods (Suchý et al., 2009), which should be respected when planning the training:

• the day after arriving as a result of arrival reaction.
• the 9th day after arriving: an individual subjective crisis, lingering until the 13th day.
• on the 15th day after arriving a third crisis sometimes appears, which may be even more severe (this crisis generally takes the form of acute depression and gradually balances out up till the nineteenth day, after which the acclimatized states stabilize)

It was found in controlled structured interviews that there are currently a number of European coaches using shortened stays at higher altitudes at a length of approximately 10–12 or 14 days (Suchý et al., 2009). The reasons for shorter sojourns are not merely socioeconomic but are also based on empirical experiences of the coaches and physiological responses discussed above.

If only very short periods are available before the altitude competition it may be recommended to arrive on the day before the competition just to check out the course, then sleep at low altitude and return to altitude the next day for the competition itself (Wilber, 2001; Foss et al., 2017).

## Conclusion and Practical Aspects

From the presented findings of various aspects of an optimal preparation for endurance competition at altitude, it may be concluded that the acclimatization process and performance recovery nearly reach a plateau after about 2 weeks at altitudes up to 4,500 m. Individual psychological conditions and appropriate diet and training are important modifiers of the preparation progress and related competition success. However, in reality preparation regimens may largely deviate from the optimum for logistical, individual and other reasons. Profound knowledge of individual physiological and psychological responses to the sojourn and training at altitude, the optimisation of diet and the consideration of individual differences will be helpful for coaches, team doctors and athletes.

## Author Contributions

All authors listed have made substantial, direct, and intellectual contribution to the work and approved it for publication.

## Conflict of Interest Statement

The authors declare that the research was conducted in the absence of any commercial or financial relationships that could be construed as a potential conflict of interest.
